# DIPG-like MYB-altered diffuse astrocytoma with durable response to intensive chemotherapy

**DOI:** 10.1007/s00381-023-05976-3

**Published:** 2023-05-11

**Authors:** Katerina Trkova, David Sumerauer, Lenka Krskova, Ales Vicha, Miroslav Koblizek, Tomas Votava, Vladimir Priban, Michal Zapotocky

**Affiliations:** 1 Prague Brain Tumor Research Group, Second Faculty of Medicine, Charles University and University Hospital Motol, V Uvalu 84, 15006 Prague 5, Czech Republic; 2 Pediatric Neurooncology Centre, University Hospital Motol, V Uvalu 84, 15006 Prague 5, Czech Republic; 3 Department of Pediatric Hematology and Oncology, Second Faculty of Medicine, Charles University Prague and University Hospital Motol, V Uvalu 84, 15006 Prague 5, Czech Republic; 4 Department of Pathology and Molecular Medicine, Second Faculty of Medicine, Charles University Prague and University Hospital Motol, V Uvalu 84, 15006 Prague 5, Czech Republic; 5 Department of Pediatrics, University Hospital in Pilsen, Alej Svobody 80, Pilsen-Lochotin, 323 00 Czech Republic; 6 Department of Neurosurgery, University Hospital in Pilsen, Alej Svobody 80, Pilsen-Lochotin, 323 00 Czech Republic

**Keywords:** MYB-altered glioma, MYB::QKI fusion, Pediatric pontine glioma, DIPG, Chemotherapy

## Abstract

Pontine gliomas represent difficult to treat entity due to the location and heterogeneous biology varying from indolent low-grade gliomas to aggressive diffuse intrinsic pontine glioma (DIPG). Making the correct tumor diagnosis in the pontine location is thus critical. Here, we report a case study of a 14-month-old patient initially diagnosed as histone H3 wild-type DIPG. Due to the low age of the patient, the MRI appearance of DIPG, and anaplastic astrocytoma histology, intensive chemotherapy based on the HIT-SKK protocol with vinblastine maintenance chemotherapy was administered. Rapid clinical improvement and radiological regression of the tumor were observed with nearly complete remission with durable effect and excellent clinical condition more than 6.5 years after diagnosis. Based on this unexpected therapeutic outcome, genome-wide DNA methylation array was employed and the sample was classified into the methylation class “Low-grade glioma, MYB(L1) altered.” Additionally, RT-PCR revealed the presence of *MYB::QKI* fusion. Taken together, the histopathological classification, molecular-genetic and epigenetic features, clinical behavior, and pontine location have led us to reclassify the tumor as a pontine MYB-altered glioma. Our case demonstrates that more intensive chemotherapy can achieve long-term clinical effect in the treatment of MYB-altered pontine gliomas compared to previously used LGG-based regimens or radiotherapy. It also emphasizes the importance of a biopsy and a thorough molecular investigation of pontine lesions.

## Introduction

Pediatric pontine gliomas represent the most challenging diagnosis in pediatric oncology due to the location and heterogeneous biology varying from indolent low-grade gliomas to aggressive diffuse intrinsic pontine glioma (DIPG) [1, 2]. DIPG is one of the deadliest tumors of childhood with a lack of curative treatment options. The majority of these tumors harbor mutations in histone H3 and are hence classified as diffuse midline gliomas H3 K27-altered. Only anecdotal cases with long-term survival have been reported so far. Making the correct tumor diagnosis in the pontine location is thus critical. Nevertheless, the majority of international institutions initiate palliative focal radiation therapy based on clinical symptoms and MRI characteristics without previous biopsy.

Here, we describe a unique case of a very young child with histone H3 wild-type DIPG that was later characterized by the presence of MYB::QKI fusion treated with radiation sparing approach.

## Case presentation

**Fig. 1:**
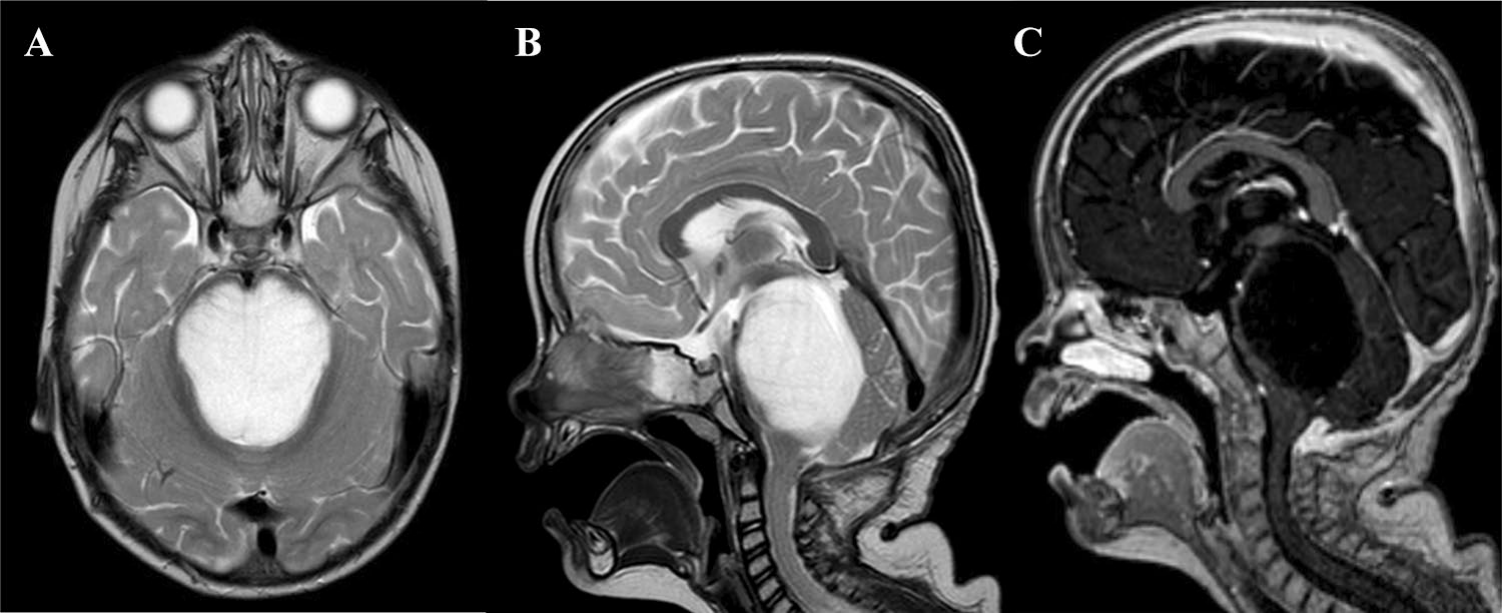
Diagnostic MRI of large pontine tumor. **A** T2-weighted axial images demonstrating pons involvement (over 66% of the diameter). **B** T2 sagittal sequence. **C** T1 sagittal after gadolinium administration displaying hypointense contrast non-enhancing tumor

**Fig. 2:**
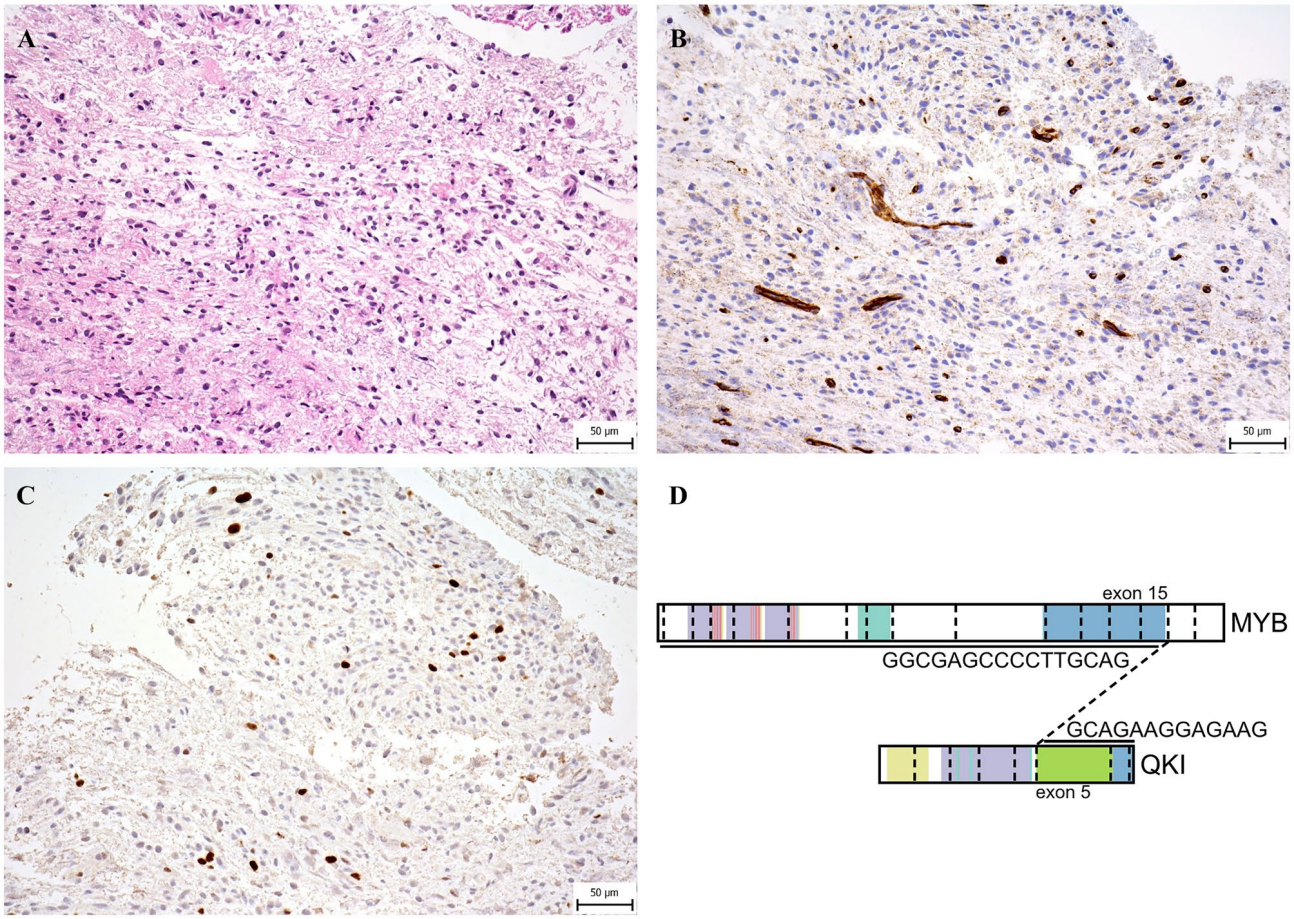
Photomicrographs of the representative tumor sections. **A** Hematoxylin and eosin–stained slide shows moderately cellular glioma with mild cytological atypia and with no obvious growth pattern. **B** Immunohistochemical staining for CD34 is negative in tumor cells and positive in endothelial cells. It highlights the absence of perivascular growth pattern. **C** Proliferative activity of tumor cells shown by Ki-67 positivity is mildly increased (generally with relatively low positivity (up to 5%), but in several hotspots clearly increased (about 10%). **D** Schematic display of *MYB::QKI* fusion transcript

At the time of diagnosis in 2016, a 14-month-old previously healthy boy presented with a 2-month history of cranial nerve palsies (n. VI and n. VII in the left side) and progressive psychomotor regression. Brain MRI revealed a T2/FLAIR hyperintense, T1 hypointense, non-enhancing extensive intraaxial posterior fossa mass centered in the pons and causing hydrocephalus. Based on MRI and clinical characteristics, the diagnosis was consistent with DIPG; however, the age at presentation was rather atypical (Fig. 1A–C). A posterior fossa craniotomy with a tumor biopsy was performed. Histopathological examination revealed a moderately cellular tumor composed of medium-sized multipolar or elongated glial fibrillary acidic protein (GFAP)–positive astrocytes with projections creating a fibrillar background. A pathologist classified this tumor as anaplastic astrocytoma grade 3 as per the 3rd version of the WHO Classification of Central Nervous Tumors from 2007 (Fig. 2A–C) [3]. Direct sequencing of the tumor tissue sample was performed at the time of diagnosis using primers described elsewhere, and neither mutations exon 15 of *BRAF* gene nor histone 3 (*H3F3A*, *HIST3B1*) was present [4, 5]. Reverse transcription PCR (RT-PCR) did not reveal any variant of the *KIAA1549::BRAF* fusion [6].

**Fig. 3:**
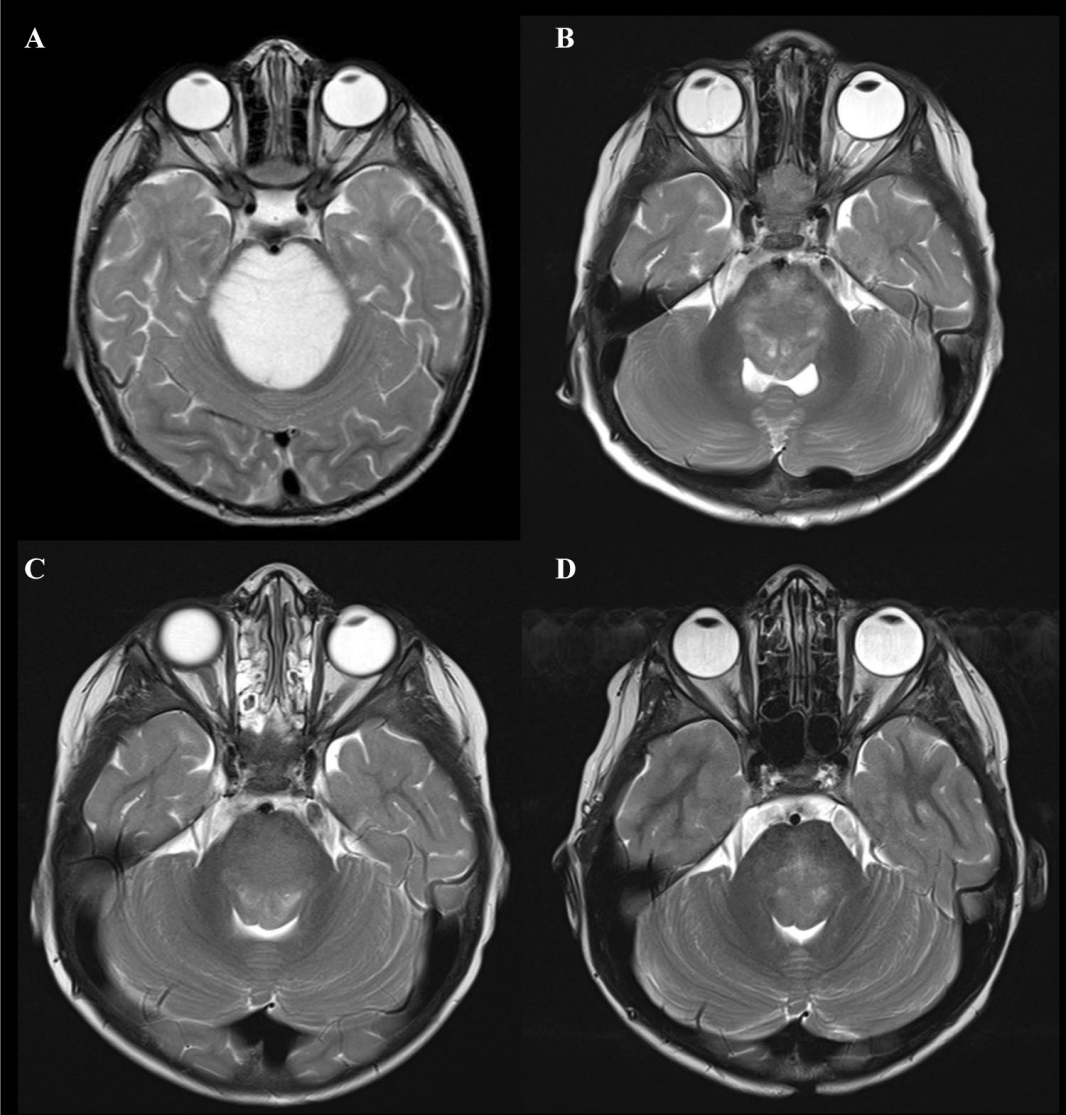
MRI demonstrating response to the therapy using T2-weighted axial images. **A** Tumor extent at the time of diagnosis. **B** Partial regression of the tumor during intensive chemotherapy (after 6 months). **C** At the end of intensive treatment and **D** continuous remission after 6.5 years from diagnosis

Due to the child’s age and better outcome of DIPG in young children (less than 3 years old) [7], intensive chemotherapy was initiated according to the German HIT-SKK-based regimen for infant high-grade glioma, consisting of 39 weeks of alternating cycles of vincristine plus cyclophosphamide, high-dose methotrexate, and carboplatin plus etoposide [8, 9]. Rapid clinical improvement followed by significant radiological partial tumor regression (> 50%) was achieved after 6 months (Fig. 3A, B). After completion of intensive chemotherapy, the patient continued with maintenance vinblastine monotherapy at a dose of 6 mg/m^2^ for a total duration of 70 weeks. Vinblastine was reduced to 5 mg/m^2^ due to hematological toxicity. At the end of the treatment, the MRI showed almost complete regression of the disease (Fig. 3C). Our patient is currently more than 6.5 years after the diagnosis with insignificant residual changes (T2-weighted images) in the pons (Fig. 3D). Clinically, he is in excellent condition without neurological deficits, living the life of a healthy child.

This favorable treatment response to chemotherapy alone and sustained durable remission were highly unlikely to occur in DIPG. Therefore, molecular analysis was expanded to evaluate true tumor biology and uncover underlying genetic aberrations. Genome-wide DNA methylation array was performed, and the sample was classified using the v12.5 version of the Heidelberg classifier into the methylation class “Low-grade glioma, MYB(L1) altered” with a calibrated score of 0.90 [10]. Consequently, we performed RT-PCR, which revealed the presence of *MYB::QKI* fusion (*ex15::ex5*) as displayed in Fig. 2D [11]. Taken together, the histopathological classification, molecular-genetic and epigenetic features, clinical behavior, and pontine location have led us to reclassify the tumor as a pontine MYB-altered glioma.

## Discussion and conclusion

According to the fifth edition of the WHO Classification of Central Nervous System Tumors from 2021, pediatric MYB-altered gliomas are split into two main groups: “Diffuse astrocytoma, MYB- or MYBL1-altered” and “Angiocentric glioma” [12]. Both tumor types are predominantly localized in the cerebral cortex [13, 14] and are associated with epilepsy, and their resection usually has curative potential [15]. Genetically, they are characterized by the presence of the *MYB* alteration; angiocentric gliomas mostly by *MYB::QKI* gene fusion; in contrast, *PCDHGA1*, *MMP16*, and *MAML* are reported fusion partner genes of *MYB/MYBL1* in diffuse astrocytomas [13].

However, only a few cases of angiocentric gliomas localized in the pons with a proven gene fusion *MYB::QKI* have been described in the literature so far [13, 16, 17]. Therapeutic LGG-based regimens used for these cases included vincristine plus carboplatin, bevacizumab, or mTOR inhibitor everolimus [16, 17]. The combined chemotherapy did not lead to a sufficient therapeutic response; only the addition of mTOR inhibitor in one patient provided stabilization of the disease [16]. Seven patients with *MYB/MYBL1*-altered brainstem gliomas were included in the single-center study from St. Jude Children’s Research Hospital. Radiation therapy was used in four out of five patients with treatment information available. All exhibited stable disease as the best response to the therapy [13]. We also reviewed the literature documenting cases of histopathologically confirmed angiocentric gliomas, but without any data on *MYB/MYBL1* alteration available [17–19]. Reported use of a LGG-based regimen consisting of vincristine and carboplatin resulted in disease progression in treated patients (*n* = 2).

Despite the fact that MYB(L1)-altered gliomas represent group of indolent low-grade tumors with reported overall survival reaching 95% at 5 years [13], tumors within brainstem location frequently require therapy. In contrast to reported treatment outcomes, our patient achieved objective response and long-term remission of the disease with radiation sparing approach. Although this is an anecdotal experience based on a single patient, it suggests that an intensive chemotherapy regimen such as HIT-SKK chemotherapy could be more effective for MYB-altered diffuse astrocytomas or angiocentric gliomas localized in the pons.

Our experience further underscores the role of biopsy in patients with brain stem tumors with the distinctive MRI appearance of DIPG in all age groups, but especially in infants, where other entities outside DMG can be encountered. As exemplified by our case, diffuse brainstem gliomas with unknown drivers should be investigated for *MYB* alterations even if they do not histologically bear an angiocentric pattern. 

## Data Availability

The datasets used and/or analyzed during the current study are available from the corresponding author on reasonable request.
